# Multi-Band Analog Radio-over-Fiber Mobile Fronthaul System for Indoor Positioning, Beamforming, and Wireless Access

**DOI:** 10.3390/s25072338

**Published:** 2025-04-07

**Authors:** Hang Yang, Wei Tian, Jianhua Li, Yang Chen

**Affiliations:** 1 Shanghai Key Laboratory of Multidimensional Information Processing, School of Communication and Electronic Engineering, East China Normal University, Shanghai 200241, China; 71215904090@stu.ecnu.edu.cn (H.Y.); wtian@cee.ecnu.edu.cn (W.T.); 2 Jiangxi Hongdu Aviation Industry Co., Ltd., Nanchang 330096, China; 13767983418@163.com; 3 Engineering Center of SHMEC for Space Information and GNSS, School of Communication and Electronic Engineering, East China Normal University, Shanghai 200241, China

**Keywords:** radio-over-fiber, remote beamforming, indoor positioning, channel state information

## Abstract

In response to the urgent demands of the Internet of Things for precise indoor target positioning and information interaction, this paper proposes a multi-band analog radio-over-fiber mobile fronthaul system. The objective is to obtain the target’s location in indoor environments while integrating remote beamforming capabilities to achieve wireless access to the targets. Vector signals centered at 3, 4, 5, and 6 GHz for indoor positioning and centered at 30 GHz for wireless access are generated centrally in the distributed unit (DU) and fiber-distributed to the active antenna unit (AAU) in the multi-band analog radio-over-fiber mobile fronthaul system. Target positioning is achieved by radiating electromagnetic waves indoors through four omnidirectional antennas in conjunction with a pre-trained neural network, while high-speed wireless communication is realized through a phased array antenna (PAA) comprising four antenna elements. Remote beamforming for the PAA is implemented through the integration of an optical true time delay pool in the multi-band analog radio-over-fiber mobile fronthaul system. This integration decouples the weight control of beamforming from the AAU, enabling centralized control of beam direction at the DU and thereby reducing the complexity and cost of the AAU. Simulation results show that the average accuracy of localization classification can reach 86.92%, and six discrete beam directions are achieved via the optical true time delay pool. In the optical transmission layer, when the received optical power is 10 dBm, the error vector magnitudes (EVMs) of vector signals in all frequency bands remain below 3%. In the wireless transmission layer, two beam directions were selected for verification. Once the beam is aligned with the target device at maximum gain and the received signal is properly processed, the EVM of millimeter-wave vector signals remains below 11%.

## 1. Introduction

Centralized radio access network (C-RAN) has become the primary wireless access network architecture for 5G due to its advantages: high capacity, high flexibility, and low latency. To meet demands for more stable and flexible communication, 5G C-RAN has evolved from a two-layer architecture consisting of a baseband unit and a remote radio unit in 4G LTE to a three-layer architecture comprising a central unit (CU), a distributed unit (DU), and an active antenna unit (AAU) [1]. The communication link between the CU and DU is referred to as the midhaul, while the link between the DU and AAU is known as the fronthaul. Mobile fronthaul (MFH) extends the fronthaul to meet the demands of mobile communication environments, imposing higher requirements on data-transmission rates and network coverage to ensure a stable and high-quality user experience.

With the advancement of the Internet of Things, an increasing number of devices, such as smart cars, low-altitude drones, and intelligent robots, rely on the high-capacity and low-latency communication capabilities of 5G. To meet the needs of these devices with regard to wireless communication, 5G and future 6G systems will expand into the millimeter-wave (mm-wave) frequency bands [2,3]. With the increase in operating frequency bands, multi-band applications, such as multi-band sensing, are emerging as important components of 5G and 6G systems beyond their basic communication functions. Considering the stronger line-of-sight (LOS) transmission characteristics and higher free-space loss of mm-waves compared to lower-frequency electromagnetic waves, the deployment density of AAU needs to be significantly increased to meet signal-coverage requirements. If traditional digital fiber transmission technology based on the common public radio interface protocol is employed to implement the MFH, its inherent bandwidth limitations and the requirement for frequency-conversion devices at the base station will significantly escalate deployment costs when mm-waves are employed. In contrast, MFH networks based on analog radio-over-fiber (ROF) technology leverage the advantages of ROF technology such as large bandwidth, low loss, and high frequency and particularly leverage its ability to directly transmit radio frequency (RF) signals over fiber. This avoids the need to deploy devices with frequency-conversion functionality and digital interfaces at the base station, thus offering better cost efficiency with higher transmission capacity.

However, analog ROF-based MFH networks may encounter dispersion and nonlinear interference during transmission, which can degrade signal-transmission performance. In comparison, digital fiber-based MFH networks can maintain high-quality signal transmission by integrating technologies such as forward error correction [4]. Therefore, in the current research literature on analog ROF, various solutions have been proposed to ensure the high performance of analog signal transmission through the optical fiber [5,6,7].

In mm-wave wireless communications, beamforming is commonly achieved through phased array antennas (PAAs), which aim to enhance signal strength in specific directions. This effectively compensates for the propagation loss of signals in free space and improves the quality of wireless communications. Traditional PAA systems rely on coaxial cables or waveguides for signal transmission; these are characterized by their large size, heavy weight, and significant signal loss. In contrast, optical PAA systems use optical fibers instead of traditional cables, offering several distinct advantages: smaller size, lighter weight, larger bandwidth, immunity to electromagnetic interference, and reduced transmission loss. Although electronic-based beamforming schemes are currently widely used, they face issues of “beam squint” and “electronic bottleneck” when operating in broadband [8].

In early optical beamforming methods, beamforming was achieved by introducing different delays to the optical signal carrying wireless signals across different optical links. In this case, multiple parallel long optical fibers are required for remote beamforming, resulting in a complex system structure. To address this issue, some remote beamforming schemes based on multi-core fibers have been proposed [9,10] that greatly simplify the transmission link. Apart from the introduction of different delays to the same optical signal using different channels, multi-wavelength technology can also be employed, leveraging the different delay characteristics of different wavelengths in the same medium to achieve different delays for signals within a single transmission channel [11,12,13]. The scheme in [11] employs a dispersion-shifted fiber, utilizing the delay induced by dispersion for remote control of the beam direction. Several single-mode-fiber (SMF)-based remote beamforming schemes have also been proposed [12,13]. In [12], the beam direction is jointly controlled by a set of fixed wavelengths and a group of variable optical delay lines. The scheme in [13] controls the beam direction solely based on the wavelength of the laser source. However, the scheme in [13] requires a large number of electro-optic modulators in the central station, leading to increased costs for the MFH network.

Although global positioning systems (GPS) perform excellently in outdoor environments, their accuracy is significantly reduced indoors due to difficulties with signal penetration through buildings [14]. Therefore, indoor positioning assisted by wireless technologies is a research hotspot and has been implemented using ultrasonic sensors, infrared systems, RF identification tags, ultra-wideband signals, Bluetooth beacons, and Wi-Fi networks [15,16].

However, apart from the commonly adopted indoor Wi-Fi devices, other wireless positioning technologies often require the deployment of dedicated hardware indoors, which is not only costly but also difficult to implement on a large scale. With the development of 5G and, in the future, 6G, the widespread deployment of AAUs in indoor environments will offer new hardware capabilities and the means for indoor positioning. Thus, combining ROF-based 5G MFH with indoor positioning represents an area of significant research importance [17,18].

In complex indoor scenarios, conventional positioning techniques face significant challenges [19]. Angle-based methods experience reduced accuracy as reflected signals distort direction-of-arrival measurements, while time-based approaches encounter obstacles to distinguishing the direct signal path from overlapping reflections. Machine learning approaches address these problems through data-driven feature learning, enabling the effective characterization of nonlinear propagation dynamics in scenarios with rich multipath components. This capability allows such methods to model complex signal behavior comprehensively, thereby enhancing the accuracy and reliability of indoor positioning systems. The data required for machine learning-based methods can be obtained via received signal strength indication (RSSI) [20,21] or channel state information (CSI) [22,23,24]. For example, in transmitting a data resource block (RB) containing multiple orthogonal frequency division multiplexing (OFDM) symbols, the RSSI-based method marks the average power of the RB as a characteristic value, while the CSI-based method involves parsing the OFDM RB, analyzing the CSI of each subcarrier after it passes through the channel, and using thus as a characteristic value to achieve positioning through intelligent algorithms.

**Figure 1:**
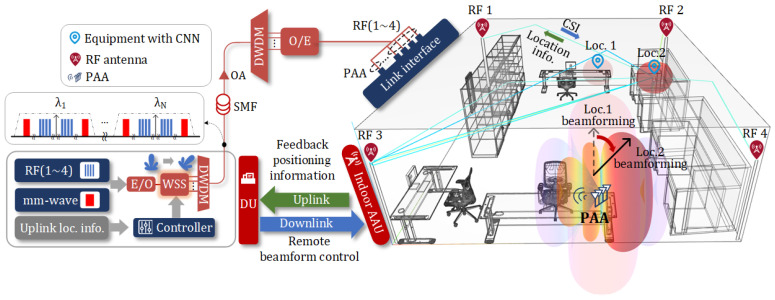
Schematic diagram and application scenario for the proposed multi-band analog ROF-MFH with remote beamforming and indoor positioning capabilities. DU, distributed unit; AAU, active antenna unit; E/O, electro-optical conversion; WSS, wavelength-selective switch; DWDM, dense wavelength division multiplexing; SMF, single-mode fiber; OA, optical amplifier; O/E electro-optical conversion; CSI, channel state information; CNN, convolutional neural network.

In this work, we propose a multi-band analog ROF-MFH with remote beamforming and indoor positioning capabilities. A schematic diagram and application scenario are illustrated in Figure 1. At the DU end, a wavelength-selective switch (WSS) is used to select a group of specific optical wavelengths carrying vector signals centered at 3, 4, 5, and 6 GHz for indoor positioning for low-speed wireless access and vector signals centered at 30 GHz for high-speed wireless access. Different wavelengths create varying delays due to dispersion when they are transmitted through SMF, forming an optical true time delay pool (OTTD-P) [13]. This allows the DU to control the beam direction of the AAU’s PAA. Additionally, low-frequency RF vector signals in OFDM format are used for indoor positioning, in addition to low-speed wireless communications. When four omnidirectional transmitting antennas are fixed in the four corners of the room and the receiving antenna is located anywhere indoors, the different wireless channels result in varying CSI values for the OFDM subcarriers. These varying CSI values are used to train a convolutional neural network (CNN) with positioning capabilities. Once the CNN is trained, it is embedded in the receiving device, enabling it to obtain its location in real time and feed this information back to the DU via the uplink from the AAU. This enables the adjustment of the mm-wave beam direction to align with the target device, thereby improving the reception quality of the mm-wave vector signal for high-speed wireless communications. This integrated architecture incorporates remote beamforming and indoor positioning technologies that can address challenges in diverse scenarios. In urban office buildings and household environments, ROF-based remote beamforming and CSI-based indoor positioning can provide precise information on target location, thereby enabling high-speed data connections. Furthermore, the architecture can also be used to enhance industrial automation and infrastructure management in commercial buildings. For rural areas, the proposed method can provide cost-effective means of target location and information transmission in applications such as greenhouse monitoring and telemedicine. Additionally, centralized beam control significantly reduces the cost of infrastructure deployment in any scenario. Simulation results show that in a 3D model of the Microwave Photonics Laboratory at East China Normal University, the average accuracy of localization classification can reach 86.92%, with six distinguished beam directions for the PAA achieved via the OTTD-P. In optical link transmission, when the received optical power (ROP) is 10 dBm, the error vector magnitudes (EVMs) of vector signals in all frequency bands remain below 3%. Regarding the wireless transmission layer, two beam directions were selected for verification. Once the beam is aligned with the target device at the highest gain and the received signal is properly processed, the EVM of mm-wave vector signals is better than 11%.

## 2. Principle

### 2.1. System Architecture and Signal Transmission

**Figure 2:**
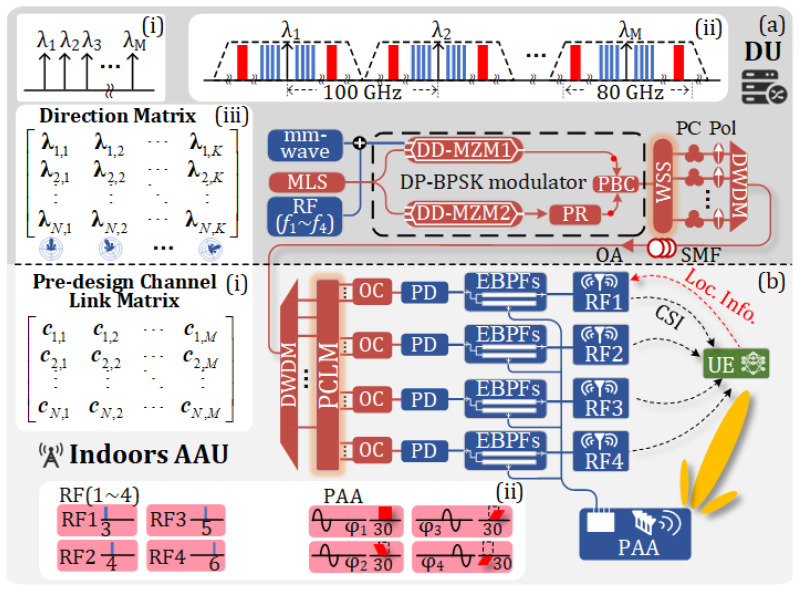
System structure of the proposed multi-band analog ROF-MFH with remote beamforming and indoor positioning capabilities. (**a**) shows the structure of the DU; (**b**) shows the structure of the AAU and its information exchange with the UE. (**a**(**i**)–**a**(**iii**)) show the schematic of the optical carriers, the schematic of the optical signal from the DP-BPSK modulator, and the beam direction matrix, respectively. (**b**(**i**),**b**(**ii**)) show the pre-designed channel link matrix and the schematic of the mm-wave vector signal and four RF vector signals, respectively. DU, distributed unit; AAU, active antenna unit; MLS, multi-wavelength laser source; DWDM, dense wavelength division multiplexing; DP-BPSK modulator, dual-polarization binary phase-shift keying modulator; DD-MZM, dual-drive Mach–Zehnder modulator; PR, polarization rotator; PBC, polarization beam combiner; PC, polarization controller; Pol, polarizer; WSS, wavelength-selective switch; SMF, single-mode fiber; OA, optical amplifier; PCLM, pre-designed channel link matrix; OC, optical coupler; PD, photodetector; EBPFs, electrical bandpass filters; RF, radio frequency; PAA, phased array antenna; CSI, channel state information, UE, user equipment.

The detailed structure of the proposed multi-band analog ROF-MFH is shown in Figure 2, while Figure 2a,b, respectively, illustrate the composition of the DU and that of the indoor AAU.

In the DU, a multi-wavelength laser source (MLS) generates multiple optical carriers with a fixed wavelength interval of Δλ. The schematic of the optical carriers is shown in Figure 2a(i). These optical carriers are injected into a dual-polarization binary phase-shift keying (DP-BPSK) modulator. One mm-wave vector signal and four RF vector signals with much lower frequencies are coupled and applied to one RF port of the upper dual-drive Mach–Zehnder modulator (DD-MZM1) in the DP-BPSK modulator. The other three RF ports of the DP-BPSK modulator have no inputs. DD-MZM1 is biased at the minimum transmission point, while DD-MZM2 is not subjected to any DC bias.

For ease of analysis, it is assumed that the optical signal injected into the DP-BPSK modulator has only one wavelength and that the RF port is injected with only one vector signal. The vector signal is expressed as $S_{\mathrm{RF}}(t)=A_{\mathrm{RF}}\cos(\omega_{\mathrm{RF}}t+\varphi_{\mathrm{RF}}(t))$, where $A_{\mathrm{RF}}$, $\omega_{\mathrm{RF}}$, and $\varphi_{\mathrm{RF}}(t)$ are the amplitude, angular frequency, and phase of the vector signal, respectively. Thus, the output signal of DD-MZM1 can be expressed as follows:

(1) $$E_{\mathrm{DD}-\mathrm{MZM}1}(t)=\frac{1}{2}\exp\left\{j\omega_{c}t+jm_{\mathrm{RF}}\cos(\omega_{\mathrm{RF}}t+\varphi_{\mathrm{RF}}(t))\right\}+\frac{1}{2}\exp(j\omega_{c}t+j\pi ),$$

where $\omega_{c}$ is the angular frequency of the optical carrier, $V_{\pi }$ is the half-wave voltage of the modulator, and $m_{\mathrm{RF}}=\pi A_{\mathrm{RF}}/V_{\pi }$ is the modulation index of the vector signal. Using the first-order Jacobi−Anger expansion, Equation (1) can be further expressed as follows:

(2) $$E_{\mathrm{DD}-\mathrm{MZM}1}(t)\approx \exp(j\omega_{c}t)\left(\frac{1}{2}[J_{0}(m_{\mathrm{RF}})-1]+jJ_{1}(m_{\mathrm{RF}})\cos(\omega_{\mathrm{RF}}t+\varphi_{\mathrm{RF}}(t))\right).$$

When the small-signal modulation condition ($m_{\mathrm{RF}}<<1$) is satisfied, Equation (2) can be further simplified as follows:

(3) $$E_{\mathrm{DD}-\mathrm{MZM}1}(t)\approx J_{1}(m_{\mathrm{RF}})\cos(\omega_{\mathrm{RF}}t+\varphi_{\mathrm{RF}}(t))\exp\left(j\omega_{c}t+j\frac{\pi }{2}\right).$$

The optical signal from DD-MZM1 and the pure optical carrier from DD-MZM2 on two orthogonal polarization directions are combined and output from the DP-BPSK modulator. The optical signal from the DP-BPSK modulator is selected by a WSS and then passes through a polarization controller (PC) and a polarizer (Pol). Through adjustment of the PC, the optical carrier and its sidebands are equally combined at the output of the Pol. The combined optical signal in this channel can be expressed as follows:

(4) $$E_{\mathrm{Pol}}(t)\approx \frac{\sqrt{2}}{2}J_{1}(m_{\mathrm{RF}})\cos(\omega_{\mathrm{RF}}t+\varphi_{\mathrm{RF}}(t))\exp\left(j\omega_{c}t+j\frac{\pi }{2}\right)+\frac{\sqrt{2}}{2}\exp(j\omega_{c}t+j\varphi ),$$

where φ is the static phase introduced by the PC. The output of the Pol corresponding to each channel is coupled at the dense wavelength division multiplexing (DWDM) multiplexer, transmitted from the DU to the AAU, and detected in a photodetector (PD) in the AAU. The photocurrent from the PD can be expressed as follows:

(5) $$i(t)=RJ_{1}(m_{\mathrm{RF}})\cos[\omega_{\mathrm{RF}}t+\varphi_{\mathrm{RF}}(t)]\cos\left(\frac{\pi }{2}-\varphi \right),$$

where *R* is the responsivity of the PD. When φ=π/2, the power of the signal in Equation (5) can reach its maximum value. Note that the fiber dispersion is not considered here for simplicity. As discussed in [25], fiber dispersion causes the signal after photodetection to experience a periodic power-fading effect. For a fixed frequency, the power fading can be eliminated by adjusting the static phase φ [25].

### 2.2. Remote Beamforming Based on OTTD-P

When multiple wavelengths are taken into consideration and the vector signal injected into DD-MZM1 contains multiple frequency bands, the vector signals modulated onto different optical wavelengths are identical. Each wavelength corresponds to a DWDM channel. The optical signal spectrum at the output of the DP-BPSK modulator is schematically shown in Figure 2a(ii). The optical carrier and sidebands in different channels enter the WSS, where wavelength selection is performed according to the desired beam direction in the AAU. The selected wavelengths are sent to the DWDM multiplexer for downlink transmission. The wavelength combinations that can achieve different beam directions can be described as a beam direction matrix (DM) of size N×K, as shown in Figure 2a(iii), where *N* and *K* represent the number of antenna elements in the PAA and the number of beam directions required by the AAU, respectively.

When the optical signal from the DWDM multiplexer is transmitted to the indoor AAU, a pre-designed channel link matrix (PCLM) composed of a DWDM demultiplexer and multiple optical couplers (OCs) is used for wavelength allocation, as shown in Figure 2b(i). The PCLM is an N×M matrix, where *N* and *M* represent the number of antenna elements in the PAA and the number of DWDM channels, respectively. In the AAU, the optical signals from the selected DWDM channels are detected in the PD array and filtered by different electrical band-pass filters (EBPFs).

The mm-wave vector signals from the four PDs are selected using four EBPFs and then respectively sent to the four elements of the PAA to implement wireless communication with the user equipment (UE). Since the four PDs output identical low-frequency RF vector signals, each containing four different RF vector signals, four EBPFs are used to select RF vector signals of different frequencies from the outputs of the four PDs. Then, the four different RF vector signals are sent to four different omnidirectional antennas. The UE receives the four different RF vector signals for indoor positioning. Additionally, low-speed wireless access can also be achieved by the RF vector signals.

The OTTD-P for remote beamforming is composed of the DM in Figure 2a(iii) and the PCLM in Figure 2b(i), which are used to control the remote beam direction and provide special passive wiring at the AAU, respectively. In the PCLM, the value $c_{N,M}$ is of bool type. When the value is 1, the DWDM channel with index *M* is connected to the antenna element numbered *N* in the PAA. On the contrary, when the output value is 0, they are not connected. The principle of remote beam-direction control involves utilizing the group delay difference induced by dispersion when optical signals of different wavelengths are transmitted through the SMF to control the phase of signals in each antenna element of the PAA at the AAU. For ease of analysis, it is assumed that the PAA in the AAU is composed of four equally spaced antenna elements and that there are *K* remotely controllable beam directions in the DU. The WSS and DWDM have *M* channels, where *M* corresponds to the number of wavelengths provided by the MLS. The wavelength interval of adjacent channels is assumed to be Δλ. There are two key points to note: first, the specific value of PCLM depends on the DM determined by the fiber dispersion; second, the DM and PCLM should be designed as reasonably as possible to ensure that when the DU adopts different wavelength-selection strategies, the PCLM at the AAU can accurately output exactly one different wavelength to each OC to which it is connected. If more than one optical wavelength is input into the OC from the PCLM under a certain strategy, additional wavelength-selection devices need to be introduced at the PCLM for wavelength selection. This modification can prevent mutual interference among different wavelengths but will complicate the system.

For the *k*-th direction, a specific set of wavelengths (four in this work) is selected from $\lambda_{1,k}$,$\lambda_{2,k}$,…,$\lambda_{N,k}$ in the DM. The delay $\Delta \tau_{i.j}$ between the *i*-th and *j*-th wavelengths after transmission through a section of SMF can be expressed as follows:

(6) $$\Delta \tau_{i,j}=(j-i)\Delta \lambda DL,$$

where *D* and *L* represent the dispersion coefficient and the length of the SMF, respectively. After the mm-wave vector signals carried by different wavelengths are converted back to the electrical domain in the AAU, the phase difference between mm-wave vector signals on the *i*-th and *j*-th wavelengths can be expressed as follows:

(7) $$\Delta \phi_{i,j}=2\pi f\Delta \tau_{i,j},$$

where *f* is the center frequency of the mm-wave vector signal. If the wavelength-selection strategies at the DU are properly designed, the mm-wave vector signals fed to different antenna elements possess different phase relations, enabling different beam directions to be achieved. Given that the beam direction is manipulated by controlling the WSS at the DU, remote beamforming is thereby implemented.

### 2.3. Indoor Positioning Based on CSI and CNN

With the implementation of OFDM technology, subcarrier-level channel measurements become accurate and efficient. By utilizing the pilots in OFDM symbols or using known sequences such as the long training field (LTF) sequence, CSI can be obtained at the receiving end. These means can achieve accurate estimation of the wireless channel and significantly improve signal-transmission quality.

The role of CSI in this work is to evaluate the channel characteristics of the communication link by describing the process of propagation of the signal from the four omnidirectional antennas to the UE and thus to obtain information about the channel. In a narrowband flat fading channel, the received signal at the UE can be expressed as follows [24]:

(8) $$\vec{Y}=\vec{H}\cdot \vec{X}+\vec{N},$$

where $\vec{Y}$ and $\vec{X}$ represent the received signal vector and the transmitted signal vector, respectively; $\vec{N}$ is the additive white Gaussian noise; and $\vec{H}$ represents the frequency response of the channel, which can be obtained from the CSI. The channel frequency response $\vec{H_{i}}$ of subcarrier *i* is a complex-valued quantity, which can be expressed as follows:

(9) $$H_{i}=\left|H_{i}\right|e^{j∠H_{i}}.$$

In Equation (9), $\left|H_{i}\right|$ and $∠H_{i}$ represent the amplitude response and phase response of subcarrier *i*, respectively.

**Figure 3:**
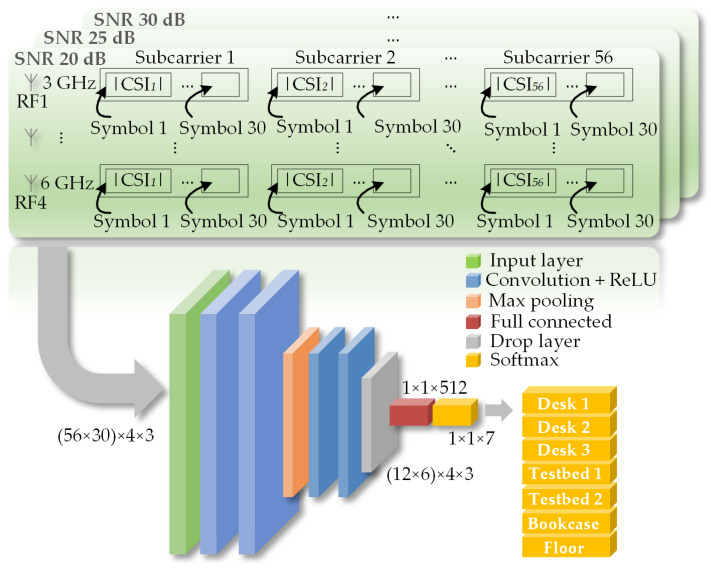
Schematic diagram of the CNN for CSI training based on the CSI information derived from the LTF sequence.

In this work, a part of OFDM symbols in one RB is used as a fixed sequence to obtain the CSI. Then, the CSI is used as input to train the CNN, which performs the positioning function. To enhance the positioning ability of the CNN, a large number of RBs need to be transmitted to form its training dataset. Meanwhile, to improve the training efficiency, only the amplitude value of the obtained CSI is used. The structure diagram of the indoor positioning CNN is shown in Figure 3. It can be seen that the obtained CSI dataset needs to be reformatted into a structure similar to an “image” so that the widely established CNN technology can be used to train a network for the positioning function.

The CNN consists of multiple layers. The input layer receives CSI data formatted as a multi-channel image. The convolutional layer conducts convolution operations to extract data features. Each convolution kernel is responsible for extracting specific types of features, thereby enhancing the network’s feature-recognition capability. The rectified linear unit (ReLU) layer applies the ReLU activation function to introduce nonlinearity for learning complex feature hierarchies. The max-pooling layer downsamples the output of the convolutional layer and the ReLU layer, reducing data dimensions and improving efficiency while retaining key features. The fully connected layer flattens the output of the previous layer and connects to all neurons, performing nonlinear combinations of features to enhance learning ability. The dropout layer randomly “drops” some neurons during training to mitigate overfitting and improve the model’s generalization ability. Finally, the Softmax layer, serving as the output layer, uses the Softmax function to convert the input into a probability distribution, providing the predicted probability for each location. In this setup, seven indoor locations are classified. Through this hierarchical structure, CNN can effectively learn complex features from CSI data, facilitating accurate classification of indoor locations.

In the CNN network, the size of the input layer is (56×30)×4×3. In this work, an RB block is defined as consisting of 100 OFDM symbols. An OFDM symbol has 64 subcarriers. Among them, the last eight subcarriers are used as pilots and the remaining 56 subcarriers carry valid data. It is worth noting that the first 30 OFDM symbols in an RB are fixed sequences used to obtain the CSI. Therefore, (56×30) represents the size of CSI carried by one RB. The value “4” refers to the four different RF vector signal frequencies of the four omnidirectional transmitting antennas, and the value “3” refers to the three different signal-to-noise ratios (SNRs). The ratio of the CNN training set to the test set is 80% to 20%, and 20% of the collected CSI data is used to validate the accuracy of the trained model.

## 3. Simulation Results

A simulation based on the setup shown in Figure 2 was carried out to verify the proposed multi-band analog ROF-MFH with remote beamforming and capabilities for indoor positioning. In the DU, the MLS generates a total of 24 wavelengths with a fixed frequency interval of 100 GHz. The frequencies of the first and last wavelengths are 193.1 THz and 195.4 THz, respectively. All the wavelengths have a linewidth of 10 MHz and a power of 20 dBm. The length of the SMF between the DU and AAU is set to 10 km. The DWDM and WSS have 24 channels and support operation at the aforementioned 24 optical wavelengths. RF vector signals with frequencies of 3, 4, 5, and 6 GHz and mm-wave vector signals with a frequency of 30 GHz are all 16-quadrature amplitude modulation (QAM) OFDM signals. The baud rates of the RF vector signal and the mm-wave vector signal are 40 Mbauds/s and 1Gbauds/s, respectively. In the simulation, the number of subcarriers of 16-QAM OFDM signals is set to 64. Among the RF vector signals for indoor positioning, 56 subcarriers are used to carry effective information and the remaining eight subcarriers are used as pilots. Each RB transmits 100 OFDM symbols. In each RB, the first 30 OFDM symbols are fixed sequences used to obtain CSI and the other 70 symbols are used to transmit effective data for low-speed wireless access.

**Figure 4:**
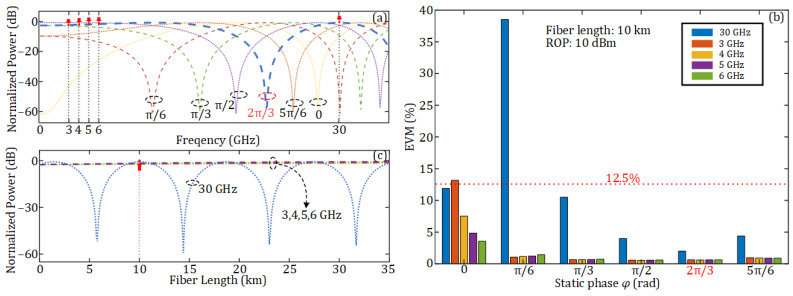
(**a**) Normalized power of the signal versus signal frequency under different static phases after transmission through a 10 km SMF; (**b**) EVM of 16-QAM OFDM signals at different frequencies and different static phases *φ* after transmission through a 10 km SMF; (**c**) normalized power of the signal versus different SMF lengths under a static phase *φ* = 2π/3.

Since double-sideband (DSB) modulation is used at the DU, fiber dispersion will introduce a periodic power fading on the recovered electrical signal at the AAU. When the SMF length between the DU and the AAU is 10 km, the normalized power of the recovered electrical signal under different carrier frequencies and different static phases *φ* is as shown in Figure 4a. It can be observed that when the static phase *φ* introduced by the PC is 2π/3, the attenuation of the 30 GHz mm-wave signal used in this work is minimized. Figure 4b illustrates the EVMs of 16-QAM OFDM vector signals at different frequencies under various static phases *φ* after transmission through the 10 km SMF. The ROP here is 10 dBm. It can be observed that the mm-wave signal exhibits the minimum EVM when the static phase *φ* = 2π/3. When *φ* = 0 and π/6, the EVM of vector signals at one frequency will exceed the 3GPP-specified minimum threshold for 16-QAM (12.5%). Therefore, *φ* is set to 2π/3 in the subsequent simulations. Besides, when *φ* = 2π/3, the four RF vector signals also have very low levels of power attenuation. Figure 4c illustrates the relationship between the normalized power and fiber length when *φ* = 2π/3 and the signal frequency is respectively set to 3, 4, 5, 6, and 30 GHz. As can be seen, when the fiber length is 10 km, the 30 GHz signal experiences no power fading and the 3, 4, 5, and 6 GHz signals only have very low levels of power fading (less than 3 dB).

**Figure 5:**
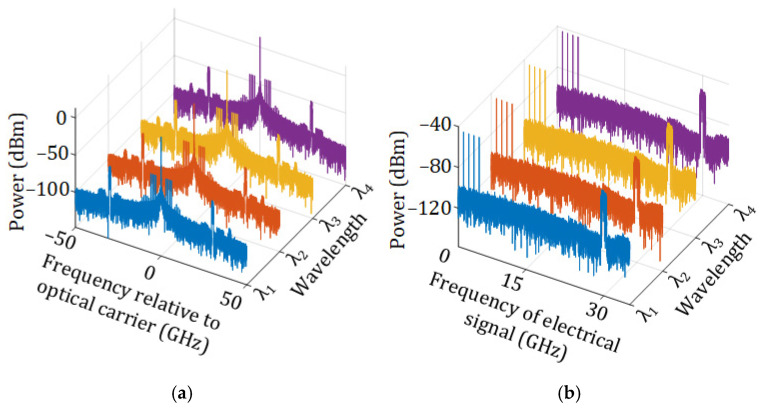
(**a**) Optical spectra of the first four wavelengths after modulation and transmission through the 10 km SMF, (**b**) the corresponding electrical spectrum after the optical signals in (**a**) are converted back to the electrical domain.

The optical spectra of the first four optical wavelengths after modulation and transmission through the 10 km SMF are shown in Figure 5a, and the corresponding electrical spectra are shown in Figure 5b. As can be seen, four RF vector signals and one mm-wave vector signal are DSB-modulated onto the first four wavelengths. After fiber transmission and optical-to-electrical conversion, they are all recovered in the electrical domain.

**Figure 6:**
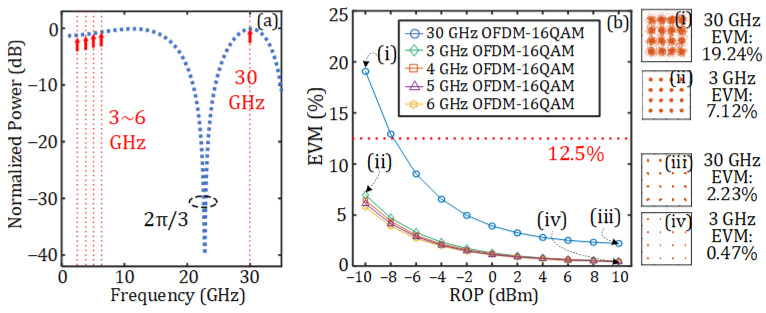
(**a**) Normalized power of the signal versus signal frequency when the static phase φ=2π/3 and the length of the SMF is 10 km. (**b**) EVM of the vector signals versus the ROP. (i–iv) show the corresponding constellation diagrams marked in (**b**).

Figure 6 further demonstrates the transmission performance of the five vector signals after they have passed through the 10 km SMF when the static phase is set to *φ* = 2π/3. Figure 6a shows the normalized power versus the signal frequency, while Figure 6b provides a detailed comparison of the relationship between the ROP and the EVM for the five vector signals. As can be seen, the four RF vector signals exhibit similar variations in EVM, with the lower frequency demonstrating a slightly inferior EVM due to its marginally greater degree of power fading, as depicted in Figure 6a. The 30 GHz mm-wave vector signal has a much worse curve due to its greater signal bandwidth and greater noise. Figure 6b(i,ii), respectively, present the constellation diagrams of signals at frequencies of 30 and 3 GHz when the ROP is −10 dBm. The EVMs are 19.24% and 7.12%, respectively. Figure 6b(iii,iv) show the constellation diagrams when the ROP is increased to 10 dBm, and the EVMs are greatly improved to 2.23% and 0.47%, respectively.

**Figure 7:**
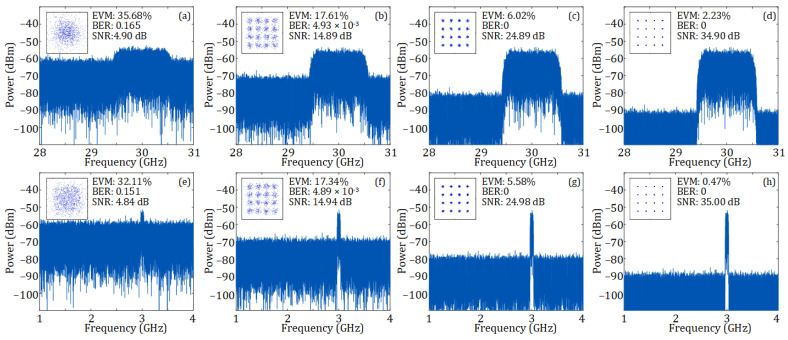
Electrical spectra at the RF port of the DP-BPSK modulator and corresponding constellation diagrams after 10 km SMF transmission and with a received optical power of 10 dBm. (**a**–**d**) show the spectra of 30 GHz input RF vector signals with SNRs of 4.90, 14.89, 24.89, and 34.90 dB, respectively; (**e**–**h**) show the spectra of 3 GHz input RF vector signals with SNRs of 4.84, 14.94, 24.98, and 35.00 dB, respectively. The insets show the corresponding constellation diagrams after fiber transmission.

Figure 7a–h, respectively, present the electrical spectra of 30 and 3 GHz 16-QAM OFDM vector signals injected into the RF port of the DP-BPSK modulator under different SNRs, along with the constellation diagrams, bit error rates (BERs), and EVMs of these signals after fiber transmission. At an SNR of around 5 dB SNR, the 30 GHz vector signal has an EVM of 35.68% and a BER of 0.165, while the 3 GHz vector signal exhibits an EVM of 32.11% and a BER of 0.151. As the SNR increases, the EVM and BER of both signals are improved. These results confirm that the analog multi-band ROF link maintains phase coherence across frequencies, as is crucial for signal integrity.

**Figure 8:**
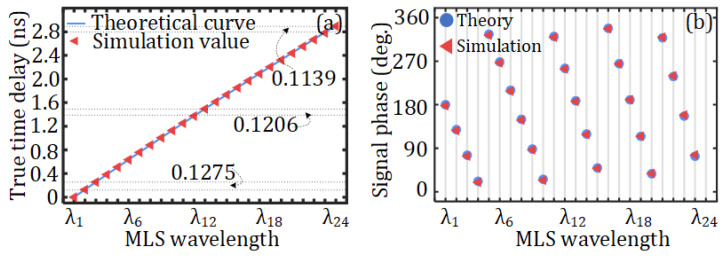
(**a**) True time delay and (**b**) corresponding phase shift for the 30 GHz mm-wave vector signal under the 24 different MLS wavelengths.

Then, the dispersion-induced time delay and phase shift using the 10 km SMF are studied. The true time delays and corresponding phase shifts for the 30 GHz mm-wave vector signal under the 24 different wavelengths are shown in Figure 8a and Figure 8b, respectively. In Figure 8a, the time delay of *λ*_1_ is used as a reference to plot the time-delay diagram for the 24 wavelengths. As can be seen from Figure 8a, the theoretical delay is in good agreement with the results obtained from the simulation.

It is worth noting that the conversion between frequency and wavelength is not linear. Therefore, when the frequency interval is fixed at 100 GHz in this study, the corresponding wavelength interval does not remain constant; the delays are thus not precisely equidistant, as marked in Figure 8a. Figure 8b presents the theoretical phase values calculated in accordance with Equation (7) and the simulated phase values, which are respectively represented by circles and triangles. By comparing Figure 8a to Figure 8b, it can be seen that the results of theory and simulation are basically consistent.

**Figure 9:**
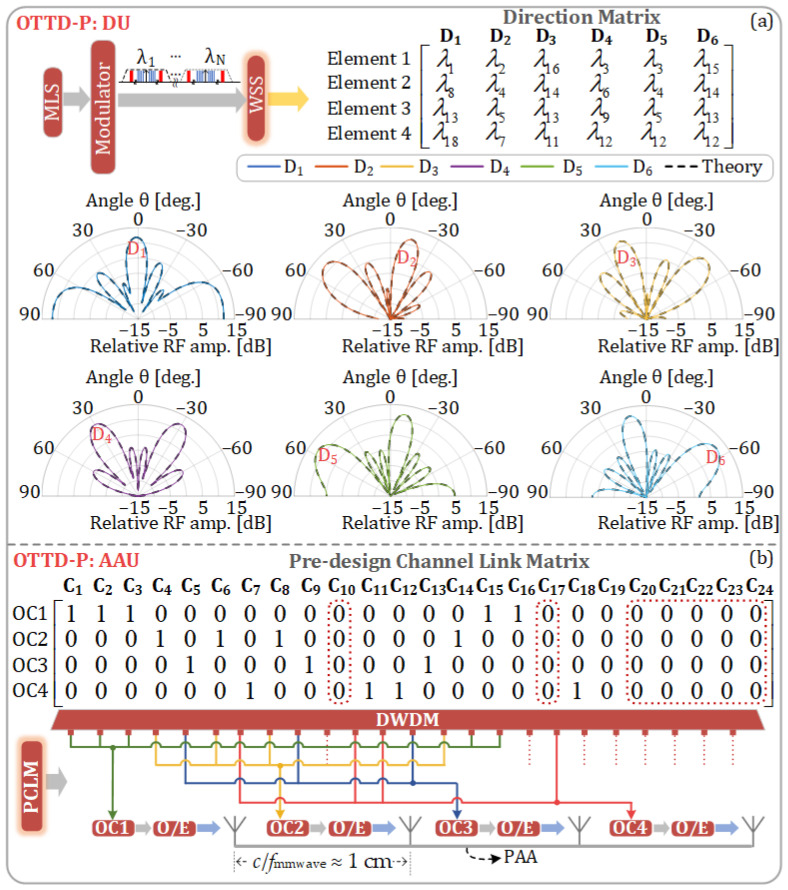
(**a**) Diagram of the OTTD-P configuration based on an equidistant four-element phased array antenna, DU-end direction matrix, and six beam directions; (**b**) AAU-end pre-designed channel link matrix and the corresponding interconnections between the DWDM demultiplexer and the four OCs.

Based on the phases corresponding to the 24 wavelengths mentioned above, four wavelengths are selected, and the phase-shifted electrical signals generated by these four selected wavelengths are fed to the four antenna elements of the PAA, respectively, so that different beam directions can be achieved. The design of the OTTD-P for an equidistant four-element PAA is illustrated in Figure 9a. Six beam directions are designed and also shown in Figure 9a. Each column of the DM in the DU represents a beam direction. The signal reaches the AAU after passing through the 10 km SMF that has a dispersion coefficient of 16 × 10^−6^ s·m^−2^. In the simulation, the center frequencies of the first and last light sources of MLS are 193.1 THz and 195.4 THz, respectively. In the DM, the matrix elements represent the wavelengths of the light sources. For example, *λ*_1_ and *λ*_24_ correspond to wavelengths centered at 1552.52 nm (193.1 THz) and 1534.24 nm (195.4 THz), respectively.

In the proposed scheme, the PAA in the AAU can achieve beam pointing in six directions. The theoretical and simulated antenna patterns are shown in the polar plots of Figure 9a, where the dashed lines represent the theoretical antenna patterns, while the solid lines represent the simulated antenna patterns. It can be observed that the antenna pattern obtained from theoretical calculation is consistent with that obtained from the simulation.

Figure 9b shows the PCLM and the corresponding interconnections in the AAU for the six beam directions in Figure 9a. The connections between the DWDM demultiplexer and the four OCs are completely determined by the PCLM. It can be observed that only 17 wavelengths are utilized in the DM of the DU and the PCLM of the AAU. Therefore, if only the six beams shown in Figure 9a are used, the DU does not require the other seven wavelengths, meaning that the MLS needs to generate only 17 wavelengths.

**Figure 10:**
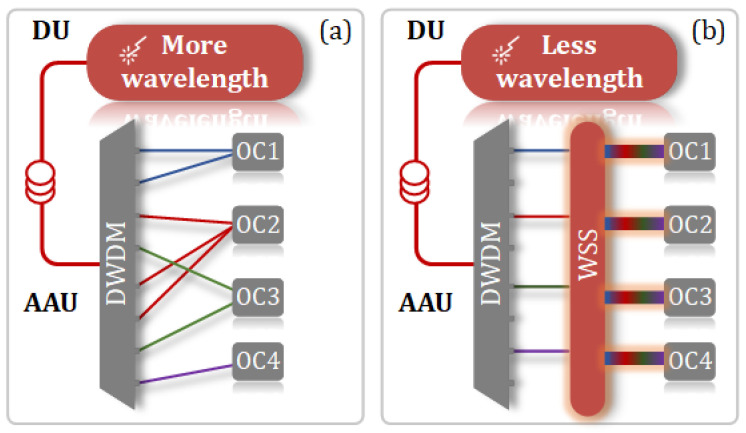
Two different strategies for wavelength selection and allocation: (**a**) using more wavelengths and passive devices in the PCLM; (**b**) using fewer wavelengths and additional WSS in the PCLM.

From the wavelength selection and allocation strategy presented in this work, as illustrated by the corresponding DM depicted in Figure 9a and PCLM depicted in Figure 9b, it can be observed that each column in the PCLM contains at most one “1”. This means that, regardless of the beam direction chosen, only one wavelength is transmitted to one OC via the PCLM. This design necessitates the use of 17 wavelengths to achieve six beam directions. The advantage of this design lies in the fact that the PCLM is entirely composed of passive components, as illustrated in Figure 10a. Initially, our strategy aims to minimize the use of lasers; hence, it is possible to achieve six beam directions while using fewer than 17 wavelengths. However, this would result in some columns of the PCLM having two or more “1” s, necessitating the use of additional WSS at the PCLM for further wavelength selection. This would convert the PCLM into an active and more costly component, as shown in Figure 10b. Given that the number of AAUs far exceeds the number of DUs, we ultimately chose the solution depicted in Figure 10a.

**Figure 11:**
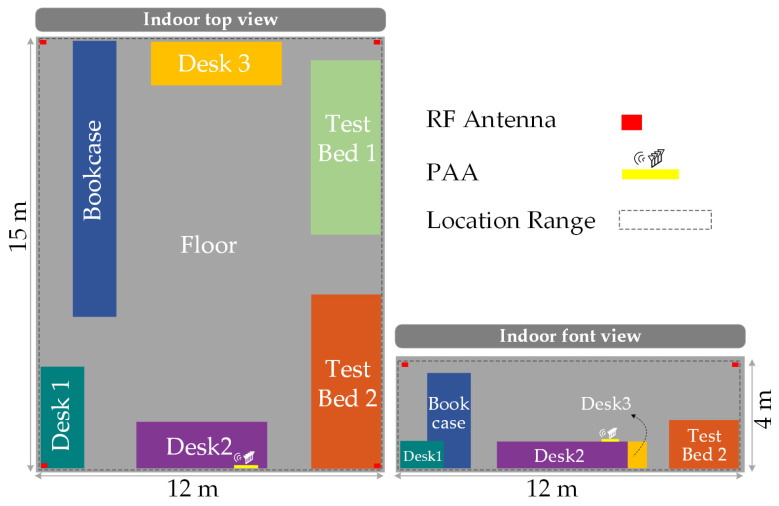
The top view and front view of the indoor simulation 3D model.

Next, a 3D model is established based on the Microwave Photonics Laboratory in East China Normal University, which is used to simulate the performance of the system proposed in this work in practical scenarios. The top view and front view of the 3D model used in the indoor simulation are shown in Figure 11.

As shown in the figure, the dimensions of the 3D model are 15 × 12 × 4 m. Based on different types of objects, the indoor space is divided into seven regions: Floor, Desk 1, Desk 2, Desk 3, TestBed 1, TestBed 2, and Bookcase. These regions will be labeled accordingly during subsequent CNN training for classification learning. The four omnidirectional antennas are distributed in the four corners of the indoor space, while the PAA is placed near the Desk 2 area. The dashed regions represent the areas designated for setting receiving antennas during CNN training. In the simulation model, the reflection materials for the floor and walls are set to concrete, while those for the desk, testbed, and bookcase are configured as metal. The channel setup considers only signals with fewer than one reflection in order to balance data complexity and analytical feasibility. It should be noted that, in this work, we focus solely on training and positioning within the aforementioned seven regions. This represents a compromise and choice made in conjunction with the training tools we use, aimed at reducing the volume of CNN training data. In practical applications in the future, the regions can be further divided into more refined regions to obtain more precise positions for the targets.

**Figure 12:**
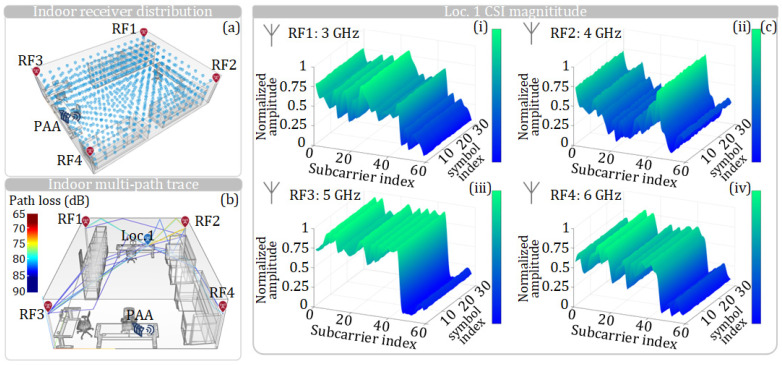
(**a**) Distribution of receiving antennas used to collect the CSI for training, with each point representing the location where a receiver should be placed to receive the four RF vector signals. (**b**) Propagation path diagram when the receiver is placed on Desk 3 (Loc. 1). (**c**) CSI-normalized amplitude responses received at Loc. 1 from the four omnidirectional antennas. (**c**(**i**)–**c**(**iv**)) show the CSI-normalized amplitude responses from the 3, 4, 5, and 6 GHz omnidirectional antennas, respectively.

To train the CNN for positioning, the CSI at different positions in the indoor 3D space needs to be obtained in advance. Figure 12a shows the distribution of receiving antennas used for collecting the CSI for training. Each point in Figure 12a represents a location where a receiver should be placed to receive the four RF vector signals and obtain the CSI at that point. For a specific location, i.e., Loc. 1, on Desk 3 in the 3D model, the propagation path diagram is shown in Figure 12b; only the direct path and the single-reflection path are considered.

When the receiver is at Loc. 1, the CSI amplitude responses of an RB transmitted by four RF omnidirectional antennas at different frequencies are shown in Figure 12c. At Loc. 1, the receiver captures and analyzes the CSI amplitude responses obtained from four RF antennas, which are then fed into the CNN shown in Figure 3 to train the network. From Figure 12c, it can be observed that, due to the different locations of the four antennas, the signals of different frequencies received at Loc. 1 from different antennas exhibit distinct CSI amplitude responses. These differences are utilized as features for CNN training. It should be noted that to obtain a CNN with higher accuracy, a large amount of CSI data must be input. There are a total of 740 positions in the 3D model, and the low-frequency RF antennas from four corners send 40 RBs to each position under SNRs of 20, 25, and 30, respectively. Regarding the hardware configuration dedicated to CNN training, we employ a GPU, specifically the NVIDIA GeForce RTX 3090 graphics card. The CNN network undergoes 100 rounds of training iterations, with the entire training process lasting 48 min. After the CNN is fully trained by using the CSI amplitude responses at different locations, the CNN can be used for positioning. Then, the CSI information obtained by a receiver, which needs to determine its position, is fed to the CNN. Through this CNN, the CSI amplitude response is matched with the pre-trained location labels and CSI amplitude response features in the network, thereby enabling the positioning of the receiver.

**Figure 13:**
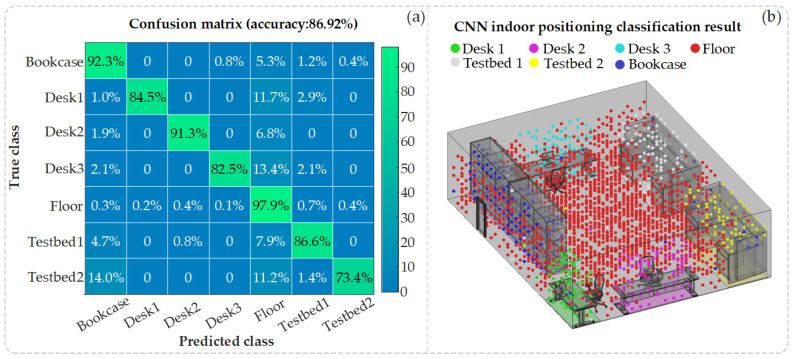
(**a**) Confusion matrix for CNN indoor positioning; (**b**) classification result diagram for CNN indoor positioning.

Figure 13a,b present, respectively, the probability confusion matrix and an illustrative diagram depicting the predicted classifications obtained following CNN training on the CSI dataset. Upon validation with the test set, the CNN model, once trained, exhibits an average location classification accuracy of 86.92%. This accuracy was determined by calculating a weighted average of the correctly predicted diagonal elements within the confusion matrix.

According to the confusion matrix, the Floor region has the highest positioning accuracy, at 97.9%. This is primarily because the Floor region covers a wide area with the most receiving points and because the space is flat, without grooves; as a result, higher CSI amplitude values are received from four directions, making the features easier to distinguish. The probability of other locations being misclassified as the Floor is also high. This phenomenon arises partly because the Floor area is large and partly because the CSI characteristics of relatively open planes at other locations are similar to the CSI characteristics of the Floor, with higher CSI amplitude values. The positioning accuracy of Bookcase ranks second. This is mainly because there are multiple grooves in Bookcase, which are easily affected by channel shadowing, resulting in low CSI amplitude values. There is also a high possibility of other locations being misclassified as Bookcases. Testbed 2 has the lowest positioning accuracy, with the highest probability of being misclassified as Floor or Bookcase. Despite the fact that Testbed 1 and Testbed 2 ought to possess comparable classification accuracy owing to their identical structural configurations, Testbed 2 is situated nearer to the indoor space’s corner (please refer to Figure 11). Therefore, Testbed 2 has a higher probability of being misclassified as Bookcase due to the lower CSI amplitude characteristics. It is worth noting that, due to the high density of receiving points in the test dataset, Figure 13b selectively presents the classification judgments of only a subset of these points for clarity. The precision of the CNN model is primarily determined by the outcomes of the confusion matrix. In practical applications, considering the complexities of indoor scenarios, as discussed in [26], a general model can be pre-trained using simulated and real-world data. As suggested in [27], this model can then be fine-tuned with new scenario-specific data, leveraging collaborative positioning strategies between simulation and actual data to enhance adaptability and efficiency and reduce training costs.

**Figure 14:**
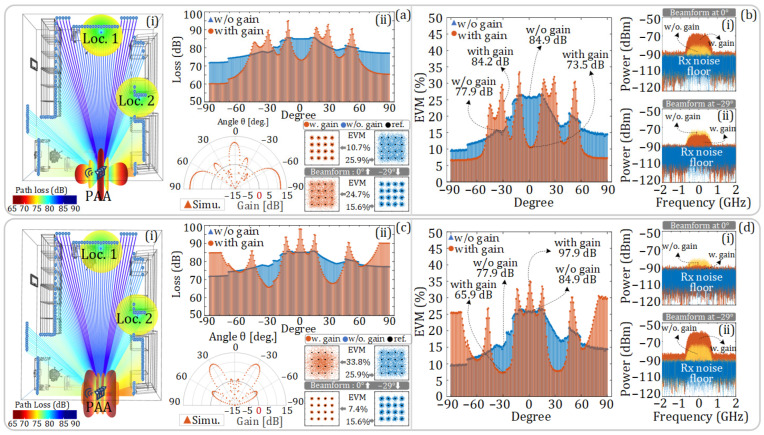
Simulation models and corresponding results. (**a**,**c**) Antenna patterns of the PAA pointing at Loc. 1 and Loc. 2, respectively, and the constellation diagrams at the receiver when the PAA is pointing at 0 degrees and −29 degrees. (**a**(**i**),**c**(**i**)) show the simulation model, (**a**(**ii**),**c**(**ii**)) show the loss of signals received by the receivers at different angles. (**b**,**d**) EVM of the signals received by the receivers at different angles. (**b**(**i**),**b**(**ii**)) show the electrical spectra when the PAA is pointing at 0 degrees and −29 degrees using the antenna pattern in (**a**), (**d**(**i**),**d**(i**i**)) show the electrical spectra when the PAA is pointing at 0 degrees and −29 degrees using the antenna pattern in (**c**).

After indoor positioning is achieved, the beam direction of the PAA can be aligned with the target through remote beamforming. As shown in Figure 14a,c, in this simulation, Loc. 1 and Loc. 2 are selected as the positions of the receivers in the 3D model, and the receiving antennas are set as omnidirectional antennas. To make the beams point to these two locations, Beam Direction 1 and Beam Direction 4 are selected from the six designed beam directions shown in Figure 9. The corresponding antenna patterns of the PAA are presented in the polar coordinates of Figure 14a and Figure 14c, respectively.

In this simulation, the SNR of the signal at the transmitter side is set to 23 dB and the noise floor of the receiver is configured as −147.02 dBm/Hz. Given that the simulation comprehensively considers the influences of beam gain, free-space loss on signal propagation, and the noise floor of the receiver, the SNR of the signal at the receiver is not constant. Besides, in this simulation, only the LOS channel is considered. In dense and complex indoor environments with frequent non-line-of-sight (NLOS) scenarios, multipath propagation and shadowing severely degrade beamforming performance. To address this issue, the proposed method can adjust the beam direction of PAA based on the positions obtained through CNN-based positioning, establishing data-transmission links through reflective paths. Considering the limited transmitting power and the complex indoor environment, a better approach is to further integrate intelligent reflecting surfaces (IRS) [28] indoors, cooperating with PAA to reconstruct the channel environment. Additionally, deep learning-based approaches [29] can be adopted for dynamic beamforming, mitigating multipath effects in complex scenarios.

Figure 14a(i),c(i) show the schematic diagram of the LOS free-space loss from –90 degrees to +90 degrees in the 3D model. In addition to Loc. 1 and Loc. 2, we have deployed multiple receivers at other locations shown in the figures. Figure 14a(ii),c(ii) show the loss of signals received by the receivers at different angles in the diagrams with and without considering the beam gain. It can be seen that the free-space loss of the signal increases with the increase in distance. After the beam gain is introduced, at the target position of 0 degrees or −29 degrees, the free-space loss is greatly compensated for.

Figure 14b and Figure 14d, respectively, show the EVM of the signals received by the receivers at different angles when the antenna pattern in Figure 14a,c are employed. Meanwhile, the electrical spectra of the received signals at 0 degrees and −29 degrees are also given. There, the blue dotted line represents the noise floor of the receiver; the red solid line represents the received signal spectrum with beam gain; and the yellow dotted line represents the received signal spectrum without beam gain.

When the receiver is located at Loc. 1, if the antenna pattern in Figure 14a is adopted, the main lobe of the PAA is aligned with Loc. 1 at 0 degrees. In this case, the loss of the signal at Loc. 1 is improved from 84.9 dB to 73.5 dB and the EVM is improved from 25.9% to 10.7%. The corresponding constellation diagrams and electrical spectra are shown in Figure 14a and Figure 14b(i), respectively. In the direction of −29 degrees, pointing at Loc. 2, the losses with and without beam gain are 84.2 dB and 77.9 dB, respectively, and the corresponding EVMs of the received signals are 24.7% and 15.6%, respectively. Since the beam gain in this direction is negative, the transmission performance of the signal is worse when the PAA is employed. The corresponding constellation diagrams and electrical spectra are shown in Figure 14a and Figure 14b(ii), respectively.

When the receiver is located at Loc. 2, if the antenna pattern in Figure 14c is adopted, the main lobe of the PAA is aligned with Loc. 2 at −29 degrees. In this case, the loss of the signal at Loc. 2 is improved from 77.9 dB to 65.9 dB, and the EVM is improved from 15.6% to 7.4%. The corresponding constellation diagrams and electrical spectra are shown in Figure 14c and Figure 14d(ii), respectively. In the direction of 0 degrees, pointing at Loc. 1, the losses with and without beam gain are 97.9 dB and 84.9 dB, respectively, and the corresponding EVMs of the received signals are 33.8% and 25.9%, respectively. The beam gain at 0 degrees is negative, so the transmission performance of the signal in this direction is worse when the PAA is employed. The corresponding constellation diagrams and electrical spectra are shown in Figure 14c and Figure 14d(i), respectively.

## 4. Conclusions

In summary, a multi-band analog ROF MFH network with indoor positioning, remote beamforming, and wireless access capabilities is presented. By integrating an OTTD-P into the ROF MFH network, remote beamforming of the PAA is achieved; this decouples the control of beamforming weights from the AAU, allowing the DU to centrally manage beam direction. At the same time, as a result of the multi-band design, the MFH network also has indoor positioning capability via four low-frequency RF vector signals. This positioning capability is realized by introducing a CNN to extract the CSI features of the channel at different locations. The location information of the target obtained from indoor positioning also provides a basis for the remote beamforming of the PAA. A simulation is conducted to verify the concept. Simulation results reveal that following a 10 km SMF transmission with an ROP of 10 dBm, the EVMs of vector signals in each frequency band remain below 3%. In indoor positioning, an average accuracy of 86.92% is achieved. Regarding beamforming, control in six directions is accomplished, and the system nearly attains full-direction indoor beam coverage. For wireless transmission, the beamforming of the PAA is verified in two directions, and after accurate positioning and appropriate signal processing, the EVM of the 30 GHz mm-wave signal passing through the wireless channel is better than 11%. This study realizes indoor positioning and remote beamforming in an ROF MFH network in which these two functions are closely related and can cooperate with each other in practical applications to achieve higher system efficiency.

## Data Availability

Data underlying the results presented in this paper are not publicly available at this time but may be obtained from the authors upon reasonable request.

## References

[1] Ji Y., Zhang J., Wang X., Yu H. (2018). Towards converged, collaborativeand co-automatic (3C) optical networks. Sci. China Inf. Sci..

[2] Hong W., Jiang Z.H., Yu C., Hou D., Wang H., Guo C., Hu Y., Kuai L., Yu Y., Jiang Z. (2021). The role of millimeter-wave technologies in 5G/6G wireless communications. IEEE J. Microw..

[3] Bazzi A., Bomfin R., Mezzavilla M., Rangan S., Rappaport T., Chafii M. (2025). Upper mid-band spectrum for 6G: Vision, opportunity and challenges. arXiv.

[4] Yao S., Chen Y.-W., Su S.-J., Alfadhli Y., Shen S., Zhang R., Zhou Q., Chang G.-K. (2020). Non-orthogonal uplink services through co-transport of D-RoF/A-RoF in mobile fronthaul. J. Lightw. Technol..

[5] Kim B.G., Bae S.H., Kim H., Chung Y.C. (2018). RoF-based mobile fronthaul networks implemented by using DML and EML for 5G wireless communication systems. J. Lightw. Technol..

[6] Ishimura S., Kao H., Tanaka K., Nishimura K., Suzuki M. (2020). SSBI-free direct-detection system employing phase modulation for analog optical links. J. Lightw. Technol..

[7] Ishimura S., Nishimura K. (2018). Direct-detection OFDM transmission using four-state chirp control with a dual-electrode MZM for dispersion compensation. IEEE Photon. J..

[8] Cao Z., Ma Q., Smolders A.B., Jiao Y., Wale M.J., Oh C.W., Wu H., Koonen A.M.J. (2016). Advanced integration techniques on broadband millimeter-wave beam steering for 5G wireless networks and beyond. IEEE J. Quantum Electron..

[9] Nagayama T., Akiba S., Tomura T., Hirokawa J. (2018). Photonics-based millimeter-wave band remote beamforming of array-antenna integrated with photodiode using variable optical delay line and attenuator. J. Lightw. Technol..

[10] Morant M., Trinidad A., Tangdiongga E., Koonen T., Llorente R. (2019). Experimental demonstration of mm-wave 5G NR photonic beamforming based on ORRs and multicore fiber. IEEE Trans. Microw..

[11] Li Y., Ghafoor S., Satyanarayana K., El-Hajjar M., Hanzo L. (2019). Analogue wireless beamforming exploiting the fiber-nonlinearity of radio over fiber-based C-RANs. IEEE Trans. Veh. Technol..

[12] Ito K., Suga M., Shirato Y., Kita N., Onizawa T. (2022). Remote beamforming scheme with fixed wavelength allocation for radio-over-fiber systems employing single-mode fiber. J. Lightw. Technol..

[13] Huang H., Zhang C., Chen C., Wu T., Huang H., Qiu K. (2018). Optical true time delay pools based centralized beamforming control for wireless base stations phased-array antennas. J. Lightw. Technol..

[14] Zafari F., Gkelias A., Leung K.K. (2019). A survey of indoor localization systems and technologies. IEEE Commun. Surveys Tuts..

[15] Wu C., Yang Z., Liu Y., Xi W. (2013). WILL: Wireless indoor localization without site survey. IEEE Trans. Parallel Distrib. Syst..

[16] Duan Y., Lam K.-Y., Lee V.C.S., Nie W., Liu K., Li H., Xue C.J. (2019). Data rate fingerprinting: A WLAN-based indoor positioning technique for passive localization. IEEE Sensors J..

[17] Farahsari P.S., Farahzadi A., Rezazadeh J., Bagheri A. (2022). A Survey on indoor positioning systems for IoT-based applications. IEEE Internet Things J..

[18] Kim J., Sung M., Cho S.-H., Won Y.-J., Lim B.-C., Pyun S.-Y., Lee J.-K., Lee J.H. (2020). MIMO-supporting radio-over-fiber system and its application in mmwave-based indoor 5G mobile network. J. Lightw. Technol..

[19] Sesyuk A., Ioannou S., Raspopoulos M. (2022). A Survey of 3D Indoor Localization Systems and Technologies. Sensors.

[20] Xue W., Qiu W., Hua X., Yu K. (2017). Improved Wi-Fi RSSI measurement for indoor localization. IEEE Sensors J..

[21] Khalajmehrabadi A., Gatsis N., Akopian D. (2017). Modern WLAN fingerprinting indoor positioning methods and deployment challenges. IEEE Commun. Surveys Tuts..

[22] Gönültas E., Lei E., Langerman J., Huang H., Studer C. (2022). CSI-based multi-antenna and multi-point indoor positioning using probability fusion. IEEE Trans. Wirel. Commun..

[23] Wu K., Xiao J., Yi Y., Chen D., Luo X., Ni L.M. (2013). CSI-based indoor localization. IEEE Trans. Parallel Distrib. Syst..

[24] Zhang Z., Lee M., Choi S. (2021). Deep-Learning-Based wi-fi indoor positioning system using continuous CSI of Trajectories. Sensors.

[25] Chen Y. (2020). A photonic-based wideband RF self-interference cancellation approach with fiber dispersion immunity. J. Lightw. Technol..

[26] Yang T., Cabani A., Chafouk H. (2021). A survey of recent indoor localization scenarios and methodologies. Sensors.

[27] Pascacio P., Casteleyn S., Torres-Sospedra J., Lohan E.S., Nurmi J. (2021). Collaborative indoor positioning systems: A systematic review. Sensors.

[28] Wu Q., Zhang R. (2019). Intelligent reflecting surface enhanced wireless network via joint active and passive beamforming. IEEE Trans. Wireless Commun..

[29] Kassir H.A., Zaharis Z.D., Lazaridis P.I., Kantartzis N.V., Yioultsis T.V., Xenos T.D. (2022). A Review of the state of the art and future challenges of deep learning-based beamforming. IEEE Access.

